# Modulation of Cytochrome P450 Metabolism and Transport across Intestinal Epithelial Barrier by Ginger Biophenolics

**DOI:** 10.1371/journal.pone.0108386

**Published:** 2014-09-24

**Authors:** Rao Mukkavilli, Sushma R. Gundala, Chunhua Yang, Shashikiran Donthamsetty, Guilherme Cantuaria, Gajanan R. Jadhav, Subrahmanyam Vangala, Michelle D. Reid, Ritu Aneja

**Affiliations:** 1 Advinus Therapeutics Limited, Bangalore, Karnataka, India; 2 Department of Biology, Georgia State University, Atlanta, Georgia, United States of America; 3 Northside Hospital Cancer Institute, Atlanta, Georgia, United States of America; 4 Department of Pathology, Emory University School of Medicine, Atlanta, Georgia, United States of America; University of Colorado Denver, United States of America

## Abstract

Natural and complementary therapies in conjunction with mainstream cancer care are steadily gaining popularity. Ginger extract (GE) confers significant health-promoting benefits owing to complex additive and/or synergistic interactions between its bioactive constituents. Recently, we showed that preservation of natural “milieu” confers superior anticancer activity on GE over its constituent phytochemicals, 6-gingerol (6G), 8-gingerol (8G), 10-gingerol (10G) and 6-shogaol (6S), through enterohepatic recirculation. Here we further evaluate and compare the effects of GE and its major bioactive constituents on cytochrome P450 (CYP) enzyme activity in human liver microsomes by monitoring metabolites of CYP-specific substrates using LC/MS/MS detection methods. Our data demonstrate that individual gingerols are potent inhibitors of CYP isozymes, whereas GE exhibits a much higher half-maximal inhibition value, indicating no possible herb-drug interactions. However, GE's inhibition of CYP1A2 and CYP2C8 reflects additive interactions among the constituents. In addition, studies performed to evaluate transporter-mediated intestinal efflux using Caco-2 cells revealed that GE and its phenolics are not substrates of P-glycoprotein (Pgp). Intriguingly, however, 10G and 6S were not detected in the receiver compartment, indicating possible biotransformation across the Caco-2 monolayer. These data strengthen the notion that an interplay of complex interactions among ginger phytochemicals when fed as whole extract dictates its bioactivity highlighting the importance of consuming whole foods over single agents. Our study substantiates the need for an in-depth analysis of hepatic biotransformation events and distribution profiles of GE and its active phenolics for the design of safe regimens.

## Introduction

The practice of integrative oncology, especially, inclusion of complementary and alternative plant-derived agents for chemotherapeutic and chemopreventive gains is steadily increasing among cancer patients'. While most of these dietary agents like spices, herbs and whole food extracts are categorized as Generally Regarded As Safe (GRAS) Agents, US FDA requires that these agents are not harmful in their intended conditions of use (i.e., consumption as therapeutic agents), are generally available and are associated with scientific evidence to establish their safety [1]. Although these dietary agents appear to be safe and well tolerated, their consumption with conventional chemotherapy and other drug regimens can be complex and can result in health complications. This is primarily because their pharmacodynamic and pharmacokinetic responses are either attenuated or enhanced depending on their metabolism and transport in physiological systems [2]. Some commonly used spices (curcumin, clove, and piperine), fruits (grape fruit, orange and cranberry) and vegetables (spinach, tomato and carrot) when administered for a long time are known to improve or fail treatments employing conventional drugs [3]. For example, interactions of drugs like ergotamine and nimodipine with grapefruit juice are known to cause gangrene or stroke [4]. On the other hand, consuming dietary supplements like St. John's wort [5], and grapefruit juice [6]–[8] with drugs like terfenadine, cyclosporine, atorvastatin and lovastatin resulted in their increased blood plasma levels resulting in undesirable/toxic side effects. Their individual effects on drug metabolizing enzymes and various uptake and efflux transporters influence the extent of interactions between these plant-based agents and conventional drugs.

The pharmacokinetic and pharmacodynamic interactions of dietary agents are due to their being substrates of Phase I and II metabolizing enzymes and drug transporters. Majorly, cytochrome P450 (CYP450) enzymes are involved in the phase I biotransformation of xenobiotics like drugs, food components, environmental toxins and other endogenous substances via their modification into corresponding metabolites [9]. CYP enzymes are highly expressed in human liver (including CYP1A2, CYP2A6, CYP2B6, CYP2C8/9/19, CYP2D6, CYP2E1 and CYP3A) and are responsible for 95% of drug metabolism [10], [11]. As the metabolism of a drug can be altered by another co-administered drug, which may prove to be clinically significant, it is important to establish the nature of interactions of a drug/herb/phytochemical i.e., if it is a substrate, inhibitor or inducer of specific CYP and other phase II conjugating enzymes and a substrate for uptake or efflux transporter(s) [9], [12]. Furthermore, inhibition of CYP enzyme activity by a dietary constituent can significantly increase the toxic effect of a drug (e.g. grapefruit juice and terfenadine) [4], which necessitates evaluation of potential drug-dietary constituent interactions. On the other hand, transport across gastrointestinal (GI) membrane also plays a key role in the biotransformation and associated activity of xenobiotics [13], [14]. Their absorption across the gut wall via passive diffusion or active uptake does not always coincide with improved bioavailability, as they may be exposed to a variety of efflux pumps including the ATP-binding cassette (ABC) transporters like P-glycoprotein (Pgp), multidrug resistance-associated protein 2 (MRP2) and breast cancer resistance protein (BCRP), which are actively involved in the transport of molecules back into the GI lumen [14]. This efflux mechanism *in vivo* is primarily responsible for poor absorption of drugs affecting their clinical development irrespective of their remarkable *in vitro* efficacy [13], [14]. It has also been reported that several food-drug interactions occur due to the ability of the food components to upregulate or inhibit the trasporter efflux pumps thus regulating the bioavailability of pharmaceutical agents [13].

Over the past few decades, substantial research has been dedicated to study the therapeutic benefits of dietary constituents, mainly plant-based food extracts and spices, which has proven to be beneficial with the discovery of disease-fighting efficacies of several whole foods and their constituent phytochemicals. Ginger is one such extensively studied spice whose therapeutic gains have encouraged its worldwide consumption [15]. Ginger extract (GE) containing a variety of active phytochemicals like 6-, 8-, and 10-gingerol, 6-paradol, 6- and 10-shogaol, zingerone, 6- and 10-gignerdione, 10-gingerdiol, 6-hydroxyshogaol, 6, 8-, and 10-dehydroshogaol and diarylheptanoids [16], [17], has been the spice of immense interest in the recent times. Extensive research on its active constituents, 6-gingerol (6G), 8-gingerol (8G), 10-gingerol (10G) and 6-shogaol (6S) has shown that GE and its components possess antioxidative, anti-inflammatory and anticancer efficacies [18]–[22]. Further, it has been reported that in humans upon consuming as low as 2 g of GE, gingerols were found to be circulating in the system (6G: 0.85±0.43 µg/mL, 8G: 0.23±0.16 µg/mL, 10G: 0.53±0.40 µg/mL, 6S: 0.15±0.12 µg/mL) albeit as their glucuronide and sulfate conjugates [23], [24].

Our laboratory is the first to identify and evaluate the anticancer activity of GE in both *in vitro* and *in vivo* prostate xenograft models [25]. We further established that the anticancer efficacy delivered by the phytochemical-rich GE is majorly due to the additive and/or synergistic interactions among its constituent biophenolics [26]. Recently, we reported that upon oral delivery of GE, its active constituents, including 6G, 8G, 10G and 6S, undergo enterohepatic recirculation for optimal therapeutic activity [27]. Also, a pharmacodynamic and pharmacokinetic comparison between the whole GE and a quasi mixture (known as Mix), formulated by combining the active GE constituents, 6G, 8G, 10G and 6S, in amounts equivalent to those present in the natural form (GE) suggested that the active phenolics collaborate with other GE constituents to effectively elicit sustained antitumor efficacy [27].

As the next obvious step of our multi-series GE study, we asked if GE and its active phenolics could modulate the hepatic biotranformation enzyme systems, particularly the CYP enzyme activity. Given that GE can potentially be included in the arsenal of GRAS agents for cancer management, it is crucial that its CYP inhibition profile is well studied to preclude any adverse drug interaction events. Here we demonstrate the effect of GE and its active phenolics on the CYP450 enzyme system and its possible biotransformation during intestinal transport. As we previously observed [27] that ginger phenolics undergo extensive conjugation in the intestine and/or liver upon oral administration of GE, we also assessed their apparent permeability to evaluate the effects of Pgp and BCRP efflux pumps on their bioavailability.

## Materials and Methods

### Cell Line and Reagents

Human liver microsomes (mixed gender, pool of 50 donors) were procured from XenoTechLLC (Kansas, USA; protein content: 20 mg/mL; catalogue number: H0610). Standard substrates and inhibitors were procured from Sigma-Aldrich, India, US Biologicals and Acros Organics. All the stable labeled internal standard(s) (IS) were from Toronto Research Chemicals, Canada. NADPH, formic acid, ammonium formate, sodium dihydrogen phosphate and disodium hydrogen phosphate, 12-well Corning Transwell filters, HBSS, HEPES, glucose, dimethyl sulfoxide (DMSO) and sodium bicarbonate were purchased from Sigma Aldrich, India. Acetonitrile (ACN) was from Merck, India. Milli-Q water (Millipore Corporation, India) was used for preparation of buffer. 96-well plates of 1 mL capacity were purchased from Axygen Scientific, USA. All gingerols and GE were isolated as previously described [27]. Caco-2, human colon carcinoma epithelial cell line was obtained from ATCC (HTB-37, Manassas, USA) and cells were used at passage number 40. Milli-cell apparatus from Millipore was used to measure the TEER.

### CYP Inhibition Assay

#### Preparation of Stock Solutions

20 mM stock solutions of 6G, 8G, 10G and 6S were prepared in ACN∶DMSO::80∶20 mixture and subsequent test dilutions of inhibitor (final concentrations of 100, 50, 25, 12.5, 6.25, 3.125, 1.563, 0.781, 0.390, 0.195 and 0.098 µM) were prepared in ACN∶DMSO::80∶20. 100 mg of ginger extract containing 3 mg of 6G, 0.680 mg of 8G, 0.770 mg of 10G and 0.590 mg of 6S was extracted with 1 mL of ACN∶DMSO (80∶20), vortex mixed for 3 min followed by sonication for 3 min and centrifuged at 5000 g for 5 min. The supernatant was collected and used for CYP inhibition assay. The highest concentration of GE used in the assay was 500 µg/mL equivalent. Stock solution for each CYP specific probe substrate was prepared in such a way that the final concentration is below the reported K_m_ value. Recommended inhibitor stock solutions were prepared in ACN∶DMSO mixture (80∶20) as given in Table S1 in File S1.

#### Assay Incubations

A microsome-buffer-substrate mixture (MBS mix) was prepared for each isozyme by pre-mixing appropriate volumes of sodium phosphate buffer (pH 7.4, 50 mM), microsomes and substrate (Table S2 in File S1). 179 µL of MBS mix was transferred to 96-well reaction plate to which 1 µL of inhibitor stock solution was added to achieve the final target inhibitor concentration. The reaction plate was pre-incubated for 10 min at 37°C followed by reaction initiation by addition of 20 µL of 10 mM NADPH solution. The reaction plate was then incubated at 37°C for a predetermined time period following which it was quenched with 200 µL ACN for all CYPs and 200 µL 1% formic acid in water∶ACN (70∶30) for CYP1A2. In all cases, the final incubations after addition of substrate and inhibitor contained 0.1% DMSO (v/v), and the total organic solvent (DMSO and ACN) content was less than or equal to 1% (v/v). The details of final substrate concentration, incubation time, microsomal protein concentration in the reaction mixtures and the metabolites monitored for each isozyme tested are presented in Table S3 in File S1. The incubations were performed in singlet for individual gingerols and in duplicate for GE along with respective positive controls.

#### Bioanalysis

All samples were processed using protein precipitation method and analyzed by employing positive (for all CYPs) and negative (for CYP2A6, 2C19 and 2E1) ionization mode in liquid chromatography tandem mass spectrometry (API4000, Applied Biosystems, USA). The peak area ratio of analyte to IS was used for calculations. An isocratic method comprising 5 mM ammonium formate and ACN (40∶60) with 0.05% formic acid was used for elution. For CYP2C19, a mobile phase consisting of 5 mM ammonium formate and ACN (30∶70) was used. The analytes and internal standards were retained on BDS Hypersil Phenyl (150×4.6 mm, 5 µ, Thermo, USA) column. A flow rate of 0.5 mL/min (CYP1A2), 0.6 mL/min (CYP2C19, CYP2E1), 0.7 mL/min (CYP2C9), 0.8 mL/min (CYP2A6, CYP3A), 1.0 mL/min (CYP2B6, CYP2C8, CYP2D6) was maintained using Shimadzu Prominence solvent delivery system (LC-20AD). The mobile phase was degassed using degasser (DGU-20A3), samples were loaded into autosampler (SIL-HTc) and the column temperature was maintained at 40°C by column oven (CTO-20A). Injection volumes for the samples were as follows: 5 µL (CYP1A2, CYP2D6, and CYP3A), 10 µL (CYP2B6, CYP2C8, and CYP2E1) and 20 µL (CYP2A6, CYP2C9, and CYP2C19). Data was collected and processed using Sciex Analyst 1.4.2. The mass transition, retention time, ionization mode for each metabolite and IS are presented in Table S4 in File S1. All the structures of inhibitors, substrates, metabolites and internal standards for each CYP enzyme are represented in Table S6 in File S1.

#### Data Analysis

The IC_50_ value was estimated from the percentage reduction in CYP activity at eleven inhibitor concentrations with respect to control. The area ratio of the metabolite in the sample without inhibitor was considered as 100%, and the percentage reduction in the CYP activity at each inhibitor concentration was determined relative to the no-inhibitor area ratio using the following equation:
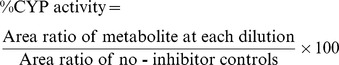
The non-linear regression model in GraphPad Prism software was used to analyze the percent CYP activity data at different concentrations and the data were fitted to the following equation and IC_50_ was calculated:
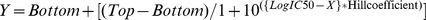
Where, X = Log concentration; Y = Response (% CYP activity)

The data was analysed using 4PL (parameter logistic model), 3PLFB (bottom fixed), 3PLFT (top fixed), 2PL (top and bottom fixed), and the relative IC_50_ was with lowest standard error was further manipulation. Absolute IC_50_ was calculated from this relative IC_50_ by using the equation below:
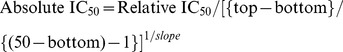



### Caco-2 Permeability Assay

#### Preparation of Cell Monolayers

Caco-2 cells were seeded on tissue culture inserts at a density of 80,000 cells/insert. The cells were maintained for 21 days in culture medium to enable differentiation and formation of the monolayer. The culture medium was changed every alternate day.

#### Preparation of Stock Solutions and Buffers

Stock solutions (1 mM) of individual gingerols were prepared in DMSO, which were then spiked into the buffer to obtain a final assay concentration of 10 µM. HBSS-HEPES buffer was prepared by dissolving 9.7 g Hank's balanced salts, 0.37 g sodium bicarbonate, 3.50 g glucose and 2.38 g HEPES in Milli-Q water (1 L). The pH of the buffer was adjusted to 7.4 using either 1N hydrochloric acid or 1N sodium hydroxide.

#### Transport Assay

Permeability of gingerols (10 µM) was determined in apical to basolateral (A-B) and basolateral to apical (B-A) directions. Transport studies were conducted between 21–25 days post seeding in 12-well Transwell inserts. Following pre-incubation in HBSS-HEPES buffer at 37°C, 5% CO_2_ for 30 min, buffer was removed and gingerols spiked buffer was added to each donor compartment (triplicate wells). Blank HBSS-HEPES buffer containing 1% DMSO was added to the receiver compartment. Samples were withdrawn from the receiver chamber at 30, 60, 90, and 120 min, and from the donor chamber at 0 and 120 min. The samples were collected in tubes containing equal volume of ACN. The withdrawn volume was replaced with buffer containing 1% DMSO. At the end of the experiment, cells were washed with ice-cold buffer and lysed with ACN to assess cell accumulation and estimate the recovery. Monolayers with transepithelial electrical resistance (TEER) above 300 Ωcm^2^ were considered for the assay and after the assay TEER values were above 300 Ωcm^2^ confirming the monolayer integrity during the experiment.

#### Bioanalysis

Samples were processed using protein precipitation method and analyzed using LC/MS/MS. A calibration curve (CC) of 5 nM to 10,000 nM range was employed to quantify the samples. 8G, 10G and 6S were eluted using a mobile phase consisting of ACN∶5 mM ammonium formate (80∶20) with 0.05% formic acid and for 6G, a mobile phase of ACN∶Milli-Q water (80∶20) with 0.1% formic acid was used. Separation was achieved using Zorbax C18 column (50×4.6 mm, 3.5 µ, Agilent) for 8G, 10G, 6S and using Hypurity C18 column (100×4.6 mm, 5 µ, Thermo) for 6G. An injection volume of 20 µL was used for all samples and a flow rate of 0.6 mL/min (6G) and 1 mL/min (8G, 10G and 6S) was used for elution. The MRM transitions (m/z) monitored were: 6G (312.3/177.1), 8G (340.0/177.1), 10G (333.0/177.1), 6S (277.2/137.1) and rolipram (276.2/208.2, Internal standard, 100 ng/mL).

#### Data Analysis

Cumulative amount of gingerols transported at each time point was plotted as function of time. The slope was used to calculate the rate of appearance of gingerols (dQ/dt).

Apparent permeability (P_app_) was calculated using the formula
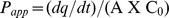
dQ/dt – rate of transport to receiver side

A - surface area of the membrane 1.12 (cm^2^)

C_0_ - initial concentration

### Statistical Analysis

CYP inhibition experiments for GE were conducted in duplicate and active phenolics in singlet. Mean IC_50_ values of GE and positive controls are presented in the result table. Permeability experiments were run in triplicates and the data are expressed as mean ± standard deviation (SD).

## Results

To ascertain the nature of interactions that ginger biophenolics undergo during Phase I metabolism when delivered individually and/or in their natural form, we incubated GE, 6G, 8G, 10G and 6S with human liver microsomes at different concentrations and evaluated their CYP enzyme inhibitory activity. The highest concentration tested for GE was 500 µg/mL (containing 15 µg/mL 6G, 3.4 µg/mL 8G, 3.9 µg/mL 10G, 3.0 µg/mL 6S). All individual components of gingerols were assessed at 100 µM equivalent to 29 µg/mL 6G, 32 µg/mL 8G, 35 µg/mL 10G and 28 µg/mL of 6S. In brief, the CYP inhibition potential of 6G was assessed at 2-fold higher concentration than that is present in GE and for 8G, 10G and 6S the CYP inhibition potential was assessed at 10-fold higher concentration than the constituent concentrations within GE. We next asked if GE and/or its active biophenolics inhibit the major membrane-bound players of CYP450 enzyme system, namely CYP1A2, CYP2A6, CYP2B6, CYP2C8, CYP2C9, CYP2C19, CYP2D6, CYP2E1 and CYP3A enzymes. The effects of GE in comparison to its active constituents on various CYP enzymes tested in this study are summarized here below.

### CYP1A2

**Figure 1:**
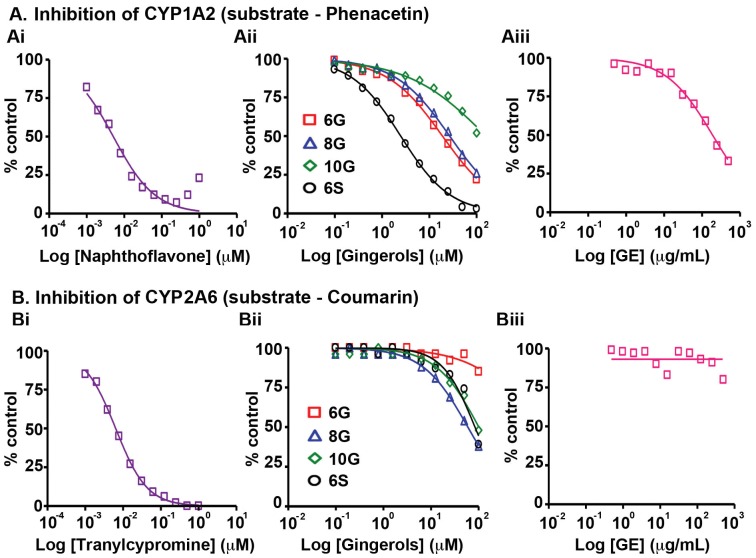
Effects of GE and its active constituents on the activity of (A) CYP1A2 with substrate, phenacetin and (B) CYP2A6 with substrate, coumarin upon incubation with human liver microsomes. The corresponding positive controls (Ai) naphthoflavone and (Bi) tranylcypromine activities were tested followed by (Aii, Bii) 6G, 8G, 10G, 6S and (Aiii, Biii) GE. Data shown are averages of duplicate experiments for GE and positive controls.

CYP1A2 isozyme is involved in the activation of procarcinogens and thus is implicated in the induction of carcinogenesis [28]. Upon incubation of human liver microsomes with GE and its active phenolics in the presence of phenacetin, CYP1A2, found in lung, oesophagus, stomach colon and primarily expressed in the liver [29], was found to be inhibited by GE with an IC_50_ of 221.5 µg/mL (Fig. 1A, Table S5 in File S1) and the corresponding 6G (5.6 µg/mL), 8G (8.8 µg/mL), 10G (>35 µg/mL) and 6S (0.7 µg/mL) IC_50_ values were similar to the concentrations of their respective counterparts present in GE (Fig. 1A, Table S5 in File S1). Nonetheless, their CYP1A2 inhibiton activity was at least ∼700–35000 fold lower than α-naphthoflavone, the positive control simulataneously tested, suggesting that in general GE and its active consituents are not potent inhibitors of CYP1A2.

### CYP2 Family

CYP2 is the largest CYP450 family in mammals including 13 subfamilies and 16 genes and is involved in the oxidation of a majority of pharmaceutical agents [29]. Most CYP2 enzymes are preferentially expressed in extrahepatic tissues, specifically in epithelia and mainly metabolize endogenous substrates [30]. GE was found to have no effect on CYP2A6 enzyme, previously known as coumarin hydroxylase, even at the highest concentration (500 µg/mL) tested, while individual gingerols and shogaol showed minimal inhibitory effect with IC_50_ value greater than 19 µg/mL (Fig. 1B, Table S5 in File S1) in the presence of coumarin. The positive control, tranylcypromine, was ∼4000 fold more effective than the test compounds with an IC_50_ value of 8.4 ng/mL.

**Figure 2:**
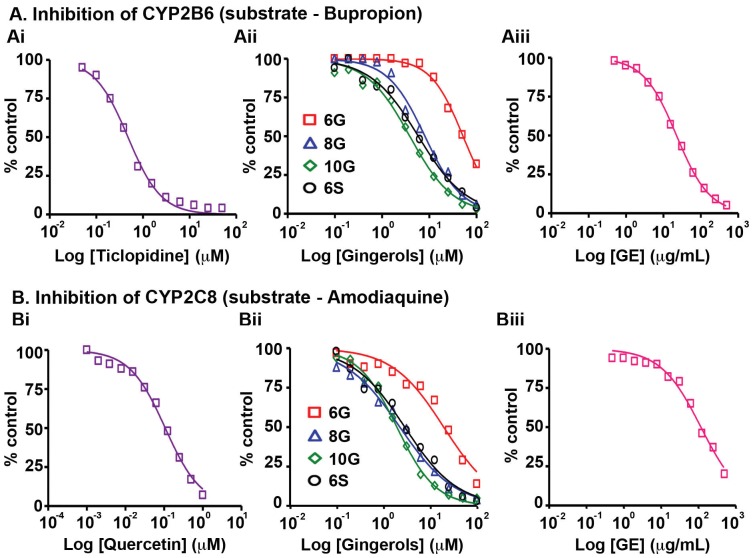
Effects of GE and its active constituents on the activity of (A) CYP2B6 with substrate, bupropion and (B) CYP2C8 with substrate, amodiaquine upon incubation with human liver microsomes. The corresponding positive controls (Ai) ticlopidine and (Bi) quercetin activities were tested followed by (Aii, Bii) 6G, 8G, 10G, 6S and (Aiii, Biii) GE. Data shown are averages of duplicate experiments for GE and positive controls.

Further analysis for CY2B6, another enzyme involved in metabolizing nicotine along with CYP2A6 [12], [29], revealed that GE showed the maximum inhibition on CYP2B6 with an IC_50_ value of 22 µg/mL (Fig. 2A, Table S5 in File S1) in the presence of bupropion. The three gingerols and shogaol exhibited ∼100–1000 fold lower inhibition of CYP2B6 with half-maximal inhibitory concentrations varying from 1.5–15 µg/mL (Fig. 2A, Table S5 in File S1) compared to ticlopidine, the positive control. Also, the inhibitory activity of GE seemed due to the contribution other partners present in GE apart from the 4 active phenolics.

**Figure 3:**
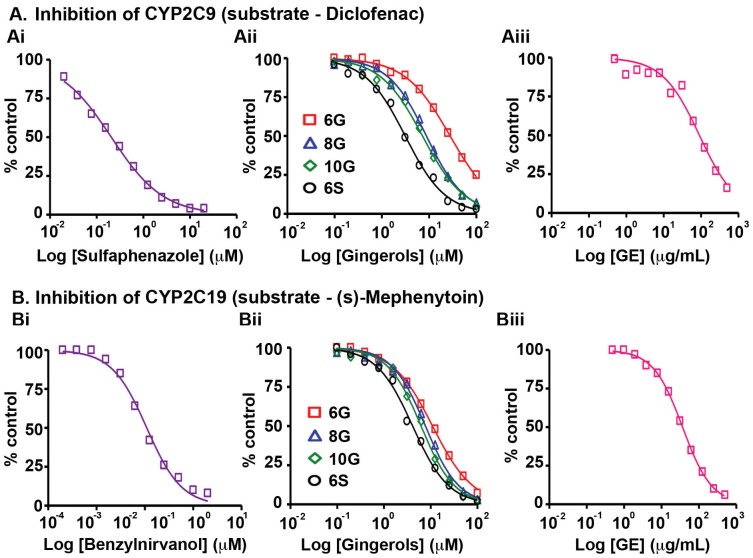
Effects of GE and its active constituents on the activity of (A) CYP2C9 with substrate, diclofenac and (B) CYP2C19 with substrate, (s)-mephenytoin upon incubation with human liver microsomes. The corresponding positive controls (Ai) sulfapenazole and (Bi) benzylnirvanol activities were tested followed by (Aii, Bii) 6G, 8G, 10G, 6S and (Aiii, Biii) GE. Data shown are averages of duplicate experiments for GE and positive controls.

Next, we evaluated the effects of GE and its active phenolics on the CYP2C subfamily including CYP2C8, CYP2C9 and CYP2C19, which are known to jointly metabolize more than 50 clinical drugs [12], [29], [31]. Detected mainly in stomach and small intestine, these enzymes colocalize in the endoplasmic reticulum [12]. GE showed an IC_50_ value of 122.5 µg/mL against CYP2C8 in the presence of amodiaquine, while 6G exhibited an IC_50_ value of 6.5 µg/mL (Fig. 2B, Table S5 in File S1). On the other hand, 8G, 10G and 6S were as potent as the positive control, quercetin (370 ng/mL), indicating a possibility of drug-drug interactions in the event of co-administration of individual gingerols and shogaols with other conventional prescription drugs. However, upon delivering these ginger biophenolics in their native form, i.e., as GE, there seemed to be neither a synergistic nor additive effect suggesting that other GE phenolics are involved in annulling the unfavorable inhibitory effect on CYP2C8 (Fig. 2B, Table S5 in File S1). GE showed an IC_50_ value 93.5 µg/mL against CYP2C9, in presence of diclofenac, the specific CYP2C9 substrate. The active ginger phytochemicals exhibited inhibitory concentrations similar to the each individual counterpart's contribution when present in GE, confirming the additive effect in inhibiting CYP2C9. The inhibitory effect of the active constituents was ∼10 to 100 fold lower than sulfaphenazole (72.7 ng/mL), the positive control (Fig. 3A, Table S5 in File S1). On the contrary, active GE phenolics showed inhibition which was ∼3 to 10 fold lower compared to ±N-3-benzylnirvanol (320 ng/mL) against CYP2C19 in the presence of (s)-mephenytoin (Fig. 3B, Table S5 in File S1). GE also exerted an inhibitory effect at equivalent concentrations as observed in case of the active constituents (35.5 µg/mL, Fig. 3B, Table S5 in File S1).

**Figure 4:**
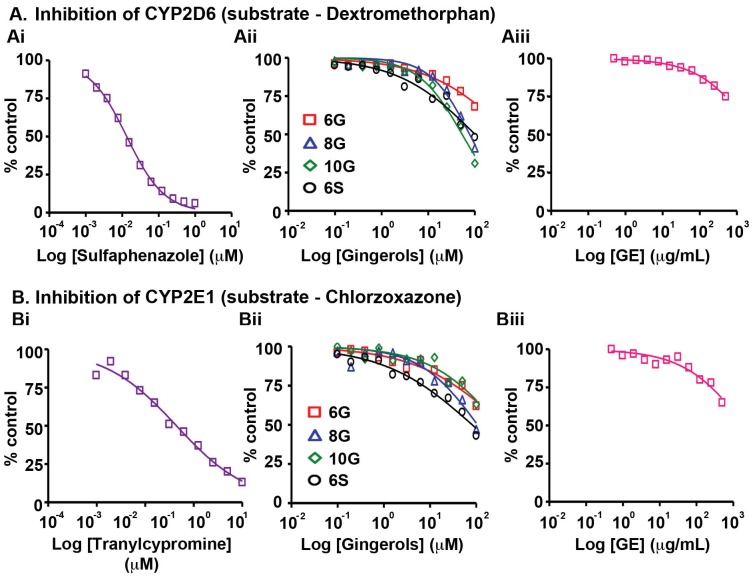
Effects of GE and its active constituents on the activity of (A) CYP2D6 with substrate, dextromethorphan and (B) CYP2E1 with substrate, chlorzoxazone upon incubation with human liver microsomes. The corresponding positive controls (Ai) sulfapenazole and (Bi) tranylcypromine activities were tested followed by (Aii, Bii) 6G, 8G, 10G, 6S and (Aiii, Biii) GE. Data shown are averages of duplicate experiments for GE and positive controls.

We next analyzed the effects of GE and its constituent active phenolics on CYP2D6, the enzyme involved in the metabolism of several endogenous substances and about 25% of marketed drugs [29], [32]. In the presence of dextromethorphan (substrate), the positive control, quinidine, inhibited CYP2D6 with an IC_50_ value of 54 ng/mL. In comparison, the active GE phenolics exhibited ∼350 to 550 fold lower activity, while GE showed no signs of inhibition at the highest concentration tested (>500 µg/mL, Fig. 4A, Table S5 in File S1). A similar effect on CYP2E1 enzyme, detected in abundance in lung, oesophagus and small intestine, was observed with GE, showing no effect even at 500 µg/mL (Fig. 4B, Table S5 in File S1). Also, the active GE constituents showed no inhibiton (∼40–70 fold lower inhibition compared to positive control tranylcypromine, IC_50_ is 579 ng/mL) (Fig. 4B, Table S5 in File S1).

**Figure 5:**
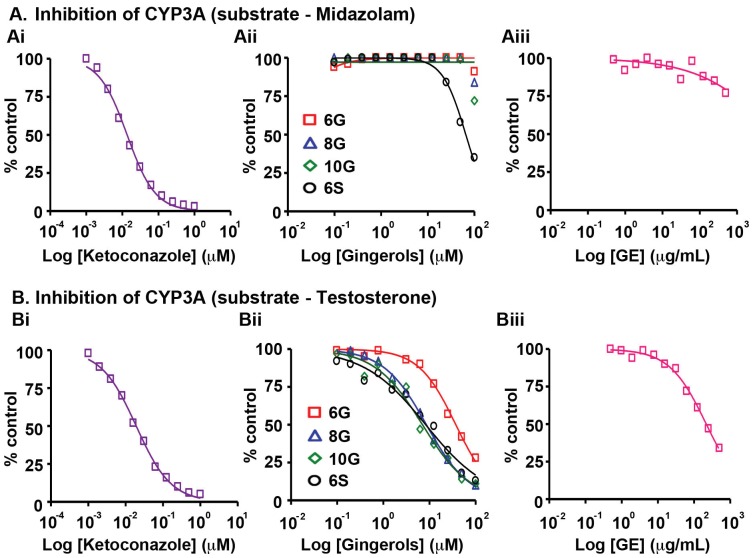
Effects of GE and its active constituents on the activity of CYP3A4 with substrates, (A) midazolam and (B) testosterone upon incubation with human liver microsomes. The corresponding positive control, (Ai and Bi) ketoconazole's inhibitory activity was tested followed by (Aii, Bii) 6G, 8G, 10G, 6S and (Aiii, Biii) GE. Data shown are averages of duplicate experiments for GE and positive controls.

Next, we analyzed the effect of GE and its active phenolics on the most abundant CYP450 enzyme, CYP3A. This enzyme highly expressed in human liver and gastrointestinal tract [33], [34] is known to metabolize more than 55% of clinical drugs [35]. Inhibition of CYP3A is reported to significantly increase the exposure of drugs, therefore resulting in an increased risk of adverse drug reactions [35]. Several dietary agents, including grape fruit juice are known to inhibit CYP3A enzymes [6]. We employed two substrates, midazolam and testosterone, which bind to different active sites on CYP3A, to determine the effect of GE and its biophenolics on enzyme activity. In the presence of midazolam, GE did not affect the enzymatic activity of CYP3A and individual constituents also showed similar profile (IC_50_ 18–35 µg/mL) (Fig. 5A, Table S5 in File S1). In presence of testosterone, the constituent biophenolics of GE showed inhibition of CYP3A activity with IC_50_ values ranging from 2.3 to 11 µg/mL (Fig. 5Bii, Table S5 in File S1). Further, the inhibition of CYP3A by GE (Fig. 5Biii, Table S5 in File S1) in the presence of testosterone confirmed the contribution of the active constituents.

The constituent phenolic compounds of GE are small organic molecules, which must be absorbed into the systemic circulation to be carried across the blood stream to target tissues and organs. Essentially, the pharmacological activity of xenobiotic compounds mainly depends on their sustained levels at the sites of action and/or their active metabolites. In order to attain sufficient effective concentrations at the target site, the orally ingested compounds have to overcome certain barriers including efflux, solubility, pH and metabolism [36], [37]. In addition, the absorption, distribution, metabolism and excretion (ADME) processes dictate their target-site concentration [38], [39]. Uptake of active molecules by enterocytes via passive diffusion or active transport is crucial for their biodistribution [2], [40]. However, favorable absorption of these phenolic compounds across the gut does not translate to their improved bioavailability and efficacy [37], [40]. Upon being absorbed by the enterocyte, the active constituents are usually presented as substrates to various efflux pumps like ATP-binding cassette (ABC) transporters, P-glycoprotein (Pgp), multidrug resistance-associated protein 2 (MRP2), and breast cancer resistance protein (BCRP), which can actively transport them back into the intestinal lumen [13], [40], [41]. Hence, we next asked if GE biophenolics serve as substrates of these efflux transporters expressed in Caco-2 cells, which will prevent them from crossing the intestinal membrane, thus leading to attenuated activity and decreased bioavailability. To this end, Caco-2 monolayers were treated with active ginger constituents (10 µM) followed by permeability assessment in apical to basolateral and basolateral to apical direction by measuring the transepithelial electrical resistance (TEER).

### GE Biophenolics Undergo Possible Biotransformation

Evaluation of *in vitro* transport profile of the active ginger constituents revealed that the apparent permeability (P_app_) of 6G, the most abundant gingerol in GE, was greater than 100 nm/sec in both apical to basal (A→B) and basal to apical (B→A) directions. The efflux ratio in this case was less than 2 confirming no possible role of efflux transporters like Pgp and BCRP in 6G's disposition. However, the recovery from A→B was less than 50% and B→A was 77% with no intracellular accumulation (<5%) (Table 1). Further, P_app_ of 8G was 53 nm/sec in A→B and 69 nm/sec in B→A direction. With an efflux ratio of 1.3, there seemed to be no interference from any efflux transporters in 8G's permeability, while its recovery in both directions was less than 60% with no cell accumulation (<1%).

**Table 1:** Permeability of active GE biophenolics across the Caco-2 semipermeable membrane.

Gingerols	6G		8G		10G		6S	
Transport (n = 3)	A→B	B→A	A→B	B→A	A→B	B→A	A→B	B→A
**P_app_ (nm/sec)**	114±15	139±16	53±8	69±6	9±7	17±6	0.71±0.14	4±2
**Efflux Ratio**	1.2		1.3		1.8		4	
**Intracellular accumulation (%)**	2	5	14	12	15	10	0	1
**Mass Balance (recovery) (%)**	49	77	36	64	22	44	4	21

The apparent permeability was calculated by measuring the drug transport across the Caco-2 monolayer. The concentration of 6G, 8G, 10G and 6S tested in this assay was 10 µM. Data presented are averages of triplicate experiments and are represented as mean ± SD.

A: apical; B: basolateral; P_app_: apparent permeability; n: number of replicates.

Interestingly, the P_app_ of 10G was lower than other active phenolics, i.e. 9 nm/sec in A→B and 17 nm/sec in B-A direction and the recovery in both directions was less than 50%, suggesting chances of metabolism with some accumulation (15%) in cells. 6S exhibited the lowest recovery, less than 25% in both directions indicating a possible biotransformation and apparent permeability values were 0.71 nm/sec and 4 nm/sec in A→B and B→A directions, respectively. However, there was almost no accumulation of 6S in cells (Table 1).

## Discussion

Phytocomplexity of ginger extract (GE) dictates the intricate synergistic or additive interactions between its constituent bioactive phenolics and thus explains the attenuation of bioactivity when single components are isolated. Essentially, the bioactivity of whole GE can perhaps be ascribed to an interaction of the biological system with multiple active compounds present in GE at low to undetectable levels. While the exact spectrum of bioactive components of GE is not fully defined, a large number of pharmacologically active compounds have been isolated and purified in various laboratories including ours. The oral bioavailability of GE and other such plant-based extracts is associated with many presystemic processes, including solubility and stability in gastrointestinal fluid, membrane permeability, transporter [e.g. P-glycoprotein (Pgp/MDR1/ABCB1)]-driven intestinal efflux, presystemic gut wall metabolism and presystemic hepatic metabolism. Suboptimal intestinal absorption of food-based extracts may occur, leading to low oral bioavailability.

With growing focus on employing alternative medicine, mainly dietary agents, as chemopreventive and chemotherapeutic agents in order to reduce the toxicity rendered by the current clinical drugs, food-drug interactions have been a major concern in regarding them as safe for consumption. Dietary supplements, when coadministered with conventional drugs, have been found to effect the latter's pharmacokinetic and pharmacodynamics interactions [6]–[8], [29], [42], [43]. Furthermore, as these interactions majorly involve metabolism and transport of drug molecules, co-administration with dietary agents could result in altering the activity of drug-metabolizing enzymes [2], [44]. Although they are “natural” and therefore considered as “safe”, little is known about the effects their constituents may have on the co-administered medication. Furthermore, it is crucial to understand the absorption, distribution, metabolism and excretion processes of such plant-derived agents to facilitate their development as dietary supplements. Polyphenols in our diet have been implicated in affecting the blood plasma concentrations of clinical drugs, thus resulting in either increased exposure or loss of their therapeutic effects [45]–[47]. Having shown the remarkable chemotherapeutic efficacy of GE, possibly due to enterohepatic recirculation of the active biophenolics [27], questions related to their impact on drug metabolizing enzymes led us to our current investigation.

Our study revealed that GE and its active constituents (6G, 8G, 10G and 6S) caused inhibition of CYP isozymes, in the order: CYP2B6>CYP2C19>CYP2C9>CYP2C8>CYP3A (testosterone as substrate)>CYP1A2 (Fig. 1A, 2A–B, 3A–B, 5B, Table S5 in File S1). GE showed the highest inhibitory effect on CYP2B6 (IC_50_ = 22 µg/mL) among all the tested CYPs, against which its active constituents showed no inhibition (Fig. 1B, 4A–B, 5A, Table S5 in File S1). This effect could be due to the partnering constituent(s) in GE other than 6G, 8G, 10G and 6S, which might be inhibitors of CYP2B6. Furthermore, the inhibitory effect of GE on CYP2C8, CYP2C9 and CYP2C19 seems to be primarily due to additive interactions among the constituent active phenolics (Table S5 in File S1). Recent reports indicate that in humans, the major components of GE (6G, 8G, 10G and 6S) are present as glucuronide and sulfate conjugates while free forms were observed in the blood plasma only upon oral administration at a high dose of 2 g per subject [23], [24]. Our current study compares the C_max_ of various gingerols reported by Zick *et al* in humans with the IC_50_ values from our CYP inhibition assay. The C_max_ of 6G was 0.85 µg/mL and the lowest IC_50_ value observed was with CYP2C19 (3.2 µg/mL) and for 8G, C_max_ was 0.23 µg/mL and lowest inhibitory concentration was with CYP2C8 (0.70 µg/mL). Similarly for 10G, C_max_ was 0.53 µg/mL and lowest IC_50_ value was with CYP2B6 (1.5 µg/mL) while C_max_ for 6S was 0.15 µg/mL and it was found to be most active against CYP1A2 (0.70 µg/mL). This comparison reveals that the highest blood plasma concentration attained for these active ginger phenolics is at least 3–4 fold lower than their respective CYP450 enzyme inhibitory concentrations *in vitro* (for CYP2C8 and CYP1A2, it was 2-fold lower). This suggests that physiologically these active GE phenolics might be incapable of modulating the Phase I metabolizing enzymes. Clearly, these plasma concentrations achieved in humans are insignificant in case of GE (Table S5 in File S1) to inhibit CYP2C9, CYP2C19, CYP2D6, CYP2E1 and CYP3A. Also, inhibition of CYP isozymes seems to be additive than synergistic and the active GE phenolics also prone to extensive conjugation in humans at intestinal level followed by liver. The overall effect of CYP inhibition by GE may be clinically irrelevant with respect to co-administered drugs that susbtrates of CYP isozymes.

Furthermore, our attemps to determine if the absorption of GE phenolics is affected by the efflux pumps across gastrointestinal membrane revealed that in the Caco-2 monolayer, permeability of 6G was highest followed by 8G, 10G and 6S (Table 1). Recovery of all the gingerols from the apical to basal side was less than 50%, which was lower than basal to apical side transport with no significant cell accumulation (Table 1). This difference could be due to extensive glucuronidation or sulfation of the ginger constituents across the Caco-2 monolayer [48], [49], supporting our previous data suggesting possible intestinal glucuronidation of 6G, 8G, 10G and 6S followed by their enterohepatic recirculation when in their native form to impart maximum efficacy to GE [27].

8G and 10G were recently shown to inhibit CYP3A4 expression, thus implicating their use in combination therapies [50]. Human intestinal microsomal content is around 10 times lower compared to the liver. While the intestinal CYPs are CYP3A (80%), CYP2C (16%), CYP2J2 (<2%) and CYP2D6 (<1%), in liver they are, CYP3A (40%), CYP2C (25%), CYP1A2 (18%), CYP2E1 (9%), CYP2A6 (6%), CYP2D6 (2%) and CYP2B6 (<1%) [51], [52]. Clearly, as no major interaction was observed in case of GE and CYP3A (Fig. 5A–B, Table S5 in File S1), the most abundant CYP in liver and intestine, and also considering the C_max_ data in humans for these ginger constituents, any major food-drug interactions involving the substrates of CYP3A are not foreseen. Further, we even speculate that only those drug molecules, which undergo conjugation reactions in the intestine and/or liver may succumb to drug-ginger interactions. This is because, it has been observed that ginger phenolics undergo extensive glucuronidation and sulfation *in vivo* [23], [27]. This is even further supported by our current observations where the recovery of gingerols was low (Table 1) across the Caco-2 cell monolayer, which is known to express uridine diphosphate glucuronosyltransferases (UGTs) and sulfotransferases (SULTs) [53]–[56], thus indicating their possible biotransformation. However, the loss in recovery of ginger constituents could also be due to lower solubility of gingerols in assay buffer and non-specific binding to assay plate, hence prompting further investigation to determine the reason for loss in recovery.

Also, as literature reports suggest that CYP1A2 is involved in the induction of carcinogenesis by metabolizing procarcinogens [29], [57], [58], the role of GE in modulating this particular enzyme requires further scrutiny. Dietary agents like cabbages, cauliflower and broccoli are known to induce the expression of CYP1A2 in humans [29], [59]. Furthermore, use of ingredients like cumin and turmeric in most of the South Asian cuisines is linked to lower activity of CYP1A2 [60]. Though our observations do not indicate the induction role of GE, our data clearly suggests that GE could inhibit CYP1A2 enzymatic activity (IC_50_ = 222 µg/mL, Table S5 in File S1) if higher concentrations of 6S were to be achieved *in vivo*. In humans, among all GE constituents, concentration of 6S was lowest (0.15 µg/mL) with propensity for both glucuronidation and sulfation. Therefore, the inhibitory activity due to 6S may be limited on CYP1A2. Hence, further exploration of GE's potential in combination with other spice constituents to prevent carcinogenesis and achieve improved anticancer efficacy seems logical.

In conclusion, our study highlights that GE and its active constituents do not modulate CYP enzyme activity, suggesting no potential prospective food-drug interactions, and thus rendering GE as safe for dietary consumption. Our observations of possible biotransformation of active ginger constituents across the Caco-2 monolayer are impelling and encourage investigation into the bioactivity of the biotransformed metabolites. Futhermore, evaluation of the accumulation of these conjugates and/or metabolites in the tumors and other tissues will aid in the design of futuristic dietary combinations/supplements to improve GE's anticancer efficacy in prostate cancer management. Our studies constitute an essential prestep before integrative medicine can be beneficial and practised to its full potential.

## Supporting Information

File S1 Table S1, Substrate and inhibitor stock solutions. Table S2, Contents in MBS (microsome-buffer-substrate) mixture. Table S3, Experimental conditions. Table S4, Mass parameters. Table S5, Inhibition of CYP activity by gingerols and positive control inhibitors in human liver microsomes. Table S6, Structures of substrates, metabolites, inhibitors and internal standards.
(DOCX)
