# Supplementary material for: Modulation of Cytochrome P450 Metabolism and Transport across Intestinal Epithelial Barrier by Ginger Biophenolics
Source: PLoS One. 2014 Sep 24;9(9):e108386. doi: 10.1371/journal.pone.0108386 (PMC4177392; doi:10.1371/journal.pone.0108386)
Supplement: File S1 — Table S1, Substrate and inhibitor stock solutions. Table S2, Contents in MBS (microsome-buffer-substrate) mixture. Table S3, Experimental conditions. Table S4, Mass parameters. Table S5, Inhibition of CYP activity by gingerols and positive control inhibitors in human liver microsomes. Table S6, Structures of substrates, metabolites, inhibitors and internal standards. (DOCX) [file pone.0108386.s001.docx]

**SUPPLEMENTARY DATA**

**Table S1: Substrate and inhibitor stock solutions**

| **Isozyme** | **Substrate** | **Stock solution**  **(mM)** | **Solvent used** | **Inhibitor** | **Stock solution**  **(mM)** |
| --- | --- | --- | --- | --- | --- |
| CYP1A2 | Phenacetin | 9 | 50% aqueous ACN | α-Naphthoflavone | 0.2 |
| CYP2A6 | Coumarin | 0.2 | ACN | Tranylcypromine | 2 |
| CYP2B6 | Bupropion | 16 | 50% aqueous ACN | Ticlopidine | 1 |
| CYP2C8 | Amodiaquin | 0.4 | 50% aqueous ACN | Quercetin | 2 |
| CYP2C9 | Diclofenac | 2 | 50% aqueous ACN | Sulfaphenazole | 4 |
| CYP2C19 | (S)-Mephenytoin | 11 | ACN | (+/-)-N-3-Benzylnirvanol | 4 |
| CYP2D6 | Dextromethorphan | 2 | ACN | Quinidine | 2 |
| CYP2E1 | Chlorzoxazone | 8 | ACN | Tranylcypromine | 20 |
| CYP3A4^a/b^ | Midazolam | 1 | ACN | Ketoconazole | 0.2 |
|  | Testosterone | 15 | ACN | Ketoconazole | 0.2 |

Table S2: Contents in MBS (microsome-buffer-substrate) mixture

| **Isozyme** | Microsomes (μL) | Buffer (μL) | Substrate (μL) |
| --- | --- | --- | --- |
| CYP1A2 | 986 | 8.4 | 5.6 |
| CYP2A6 | 990.2 | 4.20 | 5.6 |
| CYP2B6 | 991.62 | 2.78 | 5.6 |
| CYP2C8 | 993 | 1.4 | 5.6 |
| CYP2C9 | 986 | 8.4 | 5.6 |
| CYP2C19 | 980.4 | 14 | 5.6 |
| CYP2D6 | 986 | 8.4 | 5.6 |
| CYP2E1 | 991.62 | 2.78 | 5.6 |
| CYP3A-Midazolam | 988.8 | 5.6 | 5.6 |
| CYP3A-Testosterone | 986.0 | 8.4 | 5.6 |

Table S3: Experimental conditions

| **Isozyme** | **Substrate** | **Final conc. (μM)** | **Time (min)** | **Protein (mg/mL)** | **Metabolite monitored** | **Internal Standard (IS)** |
| --- | --- | --- | --- | --- | --- | --- |
| CYP1A2 | Phenacetin | 45 | 10 | 0.15 | Acetaminophen | Acetaminophen-D4 |
| CYP2A6 | Coumarin | 0.2 | 15 | 0.075 | 7’-hydroxycoumarin | 7’-hydroxycoumarin-13C3 |
| CYP2B6 | Bupropion | 16 | 20 | 0.05 | Hydroxybupropion | Hydroxybupropion-D6 |
| CYP2C8 | Amodiaquin | 0.4 | 10 | 0.025 | N-desmethylamodiaquin | N-desmethylamodiaquin-D6 |
| CYP2C9 | Diclofenac | 10 | 7 | 0.15 | 4’-hydroxydiclofenac | 4’-hydroxydiclofenac-D4 |
| CYP2C19 | (S)-Mephenytoin | 55 | 40 | 0.25 | 4’-hydroxymephenytoin | 4’-hydroxymephenytoin-D3 |
| CYP2D6 | Dextromethorphan | 10 | 7 | 0.15 | Dextrorphan | Dextrorphan-D3 |
| CYP2E1 | Chlorzoxazone | 8 | 20 | 0.05 | Hydroxychlorzoxazone | Hydroxychlorzoxazone-13C6 |
| CYP3A4^a/b^ | Midazolam | 5 | 5 | 0.10 | 1-hydroxymidazolam | 1-hydroxymidazolam-13C3 |
|  | Testosterone | 75 | 10 | 0.15 | 6β hydroxytestosterone | 6β hydroxytestosterone-D3 |

Table S4: Mass parameters

| **CYP** | **Analyte** | **MRM Transitions** | **Retention time**  **(min)** | **Ionization mode** |
| --- | --- | --- | --- | --- |
| 1A2 | Acetaminophen | 152.1/110.0 | 3.9 | Positive |
|  | Acetaminophen-D4 | 156.1/114.1 |  |  |
| 2A6 | 7’-hydroxycoumarin | 160.9/133.1 | 2.60 | Negative |
|  | 7’-hydroxycoumarin-13C3 | 163.9/135.9 |  |  |
| 2B6 | Hydroxybupropion | 256.1/238.1 | 6.71 | Positive |
|  | Hydroxybupropion-D6 | 262.2/244.1 |  |  |
| 2C8 | N-desmethylamodiaquin | 328.1/283.2 | 4.96 | Positive |
|  | N-desmethylamodiaquin-D6 | 333.1/283.2 |  |  |
| 2C9 | 4’-hydroxydiclofenac | 312.3/231.2 | 3.4 | Positive |
|  | 4’-hydroxydiclofenac-D4 | 317.1/236.1 |  |  |
| 2C19 | 4’-hydroxymephenytoin | 233.0/189.9 | 3.2 | Negative |
|  | 4’-hydroxymephenytoin-D3 | 236.1/193.1 |  |  |
| 2D6 | Dextrorphan | 258.3/157.0 | 4.1 | Positive |
|  | Dextrorphan-D3 | 261.3/157.1 |  |  |
| 2E1 | Hydroxychlorzoxazone | 183.8/119.8 | 3.3 | Negative |
|  | Hydroxychlorzoxazone-13C6 | 189.8/124.9 |  |  |
| 3A | 1-hydroxymidazolam | 342.1/203.0 | 3.5 | Positive |
|  | 1-hydroxymidazolam-13C3 | 345.1/206.1 |  |  |
| 3A | 6 β hydroxytestosterone | 305.2/269.2 | 2.2 | Positive |
|  | 6 β hydroxytestosterone-D3 | 308.1/272.5 |  |  |

Table S5: Inhibition of CYP activity by gingerols and positive control inhibitors in human liver microsomes

| **Inibitor** | \| **IC_50_ (μg/mL) for gingerols and (ng/mL) for positive controls** \| \| --- \| | | | | | | | | | |
| --- | --- | --- | --- | --- | --- | --- | --- | --- | --- | --- | --- |
|  | **CYP1A2** | **CYP2A6** | **CYP2B6** | **CYP2C8** | **CYP2C9** | **CYP2C19** | **CYP2D6** | **CYP2E1** | **CYP3A4^a^** | **CYP3A4^b^** |
| GE | 221.5 | >500 | 22 | 122.5 | 93.5 | 35.5 | >500 | >500 | >500 | 212.5 |
| 6-Gingerol | 5.6 | >29 | 15 | 6.5 | 8.2 | 3.2 | >29 | >29 | >29 | 11 |
| 8-Gingerol | 8.8 | 19 | 2.6 | 0.7 | 2.8 | 2.6 | 24 | 30 | >32 | 2.6 |
| 10-Gingerol | >35 | 34 | 1.5 | 0.7 | 2.6 | 2.3 | 19 | >35 | >35 | 2.5 |
| 6-Shogaol | 0.7 | 23 | 1.6 | 0.8 | 0.9 | 1.1 | 24 | 21 | 18 | 2.3 |
| α-Naphthoflavone | 1.2 | - | - | - | - | - | - | - | - | - |
| Tranylcypromine | - | 8.4 | - | - | - | - | - | - | - | - |
| Ticlopidine | - | - | 11.3 | - | - | - | - | - | - | - |
| Quercetin | - | - | - | 370.2 | - | - | - | - | - | - |
| Sulfaphenazole | - | - | - | - | 72.7 | - | - | - | - | - |
| (+/-)-N-3-Benzylnirvanol | - | - | - | - | - | 319.6 | - | - | - | - |
| Quinidine | - | - | - | - | - | - | 54.1 | - | - | - |
| Tranylcypromine | - | - | - | - | - | - | - | 579.3 | - | - |
| Ketoconazole^a^ | - | - | - | - | - | - | - | - | 5.3 | - |
| Ketoconazole^b^ | - | - | - | - | - | - | - | - | - | 7.4 |

^a^Midazolam, ^b^Testosterone; Stock solutions of gingerols were made equivalent to 100 μM

**Table S6:** **Structures of substrates, metabolites, inhibitors and internal standards**

| **Isozyme** | **Parent** | **Metabolite** | **Inhibitor** | **Internal Standard (IS)** |
| --- | --- | --- | --- | --- |
| **CYP1A2** |   Phenacetin |   Acetaminophen |   α-Naphthoflavone |   Acetaminophen-D4 |
| **CYP2A6** |   Coumarin |   7-hydroxycoumarin |   Tranylcypromine |   7-hydroxy coumarin-13C3 |
| **CYP2B6** |   Bupropion |   Hydroxybupropion |   Ticlopidine |   Hydroxybupropion-D6 |
| **CYP2C8** |   Amodiaquine |   N-desethylamodiaquine |   Quercetin |   N-desethylamodiaquine-D5 |
| **CYP2C9** |   Diclofenac |   4-hydroxydiclofenac |   Sulfaphenazole |   4-hydroxy diclofenac-D4 |
| **CYP2C19** |   (S)-Mephenytoin |   4-hydroxymephenytoin |   (±)-N-3-Benzylnirvanol |   4-hydroxy mephenytoin-D3 |
| **CYP2D6** |   Dextromethorphan |   Dextrorphan |   Quinidine |   Dextrorphan-D3 |
| **CYP2E1** |   Chlorzoxazone |   6-hydrxoychlorzoxazone |   Tranylcypromine |   6-hydrxoychlorzoxazone-13C6 |
| **CYP3A4** |   Midazolam |   1-hydroxy midazolam |   Ketoconazole |   1-hydroxy midazolam-13C3 |
| **CYP3A4** |   Testosterone |   6-β hydroxytestosterone |   Ketoconazole |   6-β-hydroxytestosterone-D3 |
